# Barriers to Psychosocial Support and Quality of Life for Patients With Inflammatory Bowel Disease: A Survey Study

**DOI:** 10.1093/crocol/otaa068

**Published:** 2020-08-24

**Authors:** Rebecca Lawrence, Cuckoo Choudhary

**Affiliations:** 1 Sidney Kimmel College of Medicine, Thomas Jefferson University, Philadelphia, Pennsylvania, USA; 2 Division of Gastroenterology and Hepatology, Department of Medicine, Sidney Kimmel Medical College, Thomas Jefferson University, Philadelphia, Pennsylvania, USA

**Keywords:** IBD, quality of life, barrier, psychotherapy

## Abstract

**Background:**

Psychological comorbidities are common among people with inflammatory bowel disease (IBD) and are associated with worse disease outcomes. Evidence-based psychotherapy is an effective means to increase psychosocial support. This study aimed to identify the barriers to attending psychotherapy.

**Methods:**

This electronic survey study included a demographic, quality of life, and barriers to psychotherapy questionnaire. Quality of life was assessed using the Short Inflammatory Bowel Disease Questionnaire (SIBDQ). Barriers were assessed using the Perceived Barriers to Psychological Treatments scale (PBPT). Linear regression was used to identify participant characteristics associated with higher PBPT scores.

**Results:**

One hundred eighty-seven participants completed the study. Fifty-eight percent of participants had ≥1 significant barrier. Time (28%), knowledge about the availability of services (25%), and cost (19%) were the most common barriers. Least common were stigma (14%), lack of motivation (12%), and emotional concerns (7%). Lower SIBDQ scores, being male, not being full-time employed, having Crohn disease, and being in disease remission were associated with higher PBPT scores.

**Conclusions:**

Knowledge about the availability of services, time constraints, and cost are the leading barriers to psychotherapy among people with IBD. Care providers should develop a network of psychotherapists available to those with IBD. Being male and not being full-time employed may be risk factors for greater barriers. Further research is needed on barriers among groups underrepresented in this study and on novel psychotherapy solutions, like telehealth and low-cost options.

## INTRODUCTION

Inflammatory bowel disease (IBD) is a complex, chronic disease that impacts the psychosocial elements of people’s lives. Psychiatric conditions are colloquially described as an extraintestinal manifestation of IBD.^1^ People with IBD have psychological illnesses, particularly depression and anxiety, at rates almost twice that of the general population.^2^ Having psychological comorbidities are associated with worse disease outcomes including increased hospitalizations, disease severity, rates of surgery, and reliance upon medication for disease control.^3,4^

Many studies and initiatives have called for increased attention to the psychological health of people with IBD.^1,5,6^ Several studies have explored coping strategies and psychosocial interventions. Adaptive coping strategies, like using support systems and directly addressing a problem, are associated with better disease outcomes and higher quality of life.^7,8^ Additionally, people have been found to benefit from interventions that increase psychosocial support.^9^ People with IBD tend to value psychotherapy over medications for psychological healthcare.^6^ However, even when recommended, most people do not initiate psychological therapy.^10^ Given the increased risk for psychosocial comorbidities and the potential benefit toward quality of life and disease outcome, it is worth investigating the barriers to psychosocial support among people with IBD. Identifying the most common barriers to psychosocial support and the demographic or health characteristics associated with greater barriers will allow providers to more effectively counsel and coordinate care for people who suffer from the psychosocial effects of IBD.

This study has 3 aims:

1. Identify the barriers to mental healthcare among people with IBD.
2. Assess the relationships among barriers to mental healthcare, quality of life, and demographic and health characteristics in people with IBD.
3. Offer institutional suggestions on ways to reduce barriers to mental healthcare.

## METHODS

### Subject Recruitment

This study involved an electronic survey. Eligible participants were identified by reviewing the patients seen over a 6-month period at 2 urban Gastroenterology & Hepatology clinics. Eligibility criteria included having IBD diagnosed by a gastroenterologist, being at least 18 years of age, being English literate, and having an email address listed in the medical records. Once identified, potential participants were sent an email that contained a brief explanation of the study, an electronic consent form, and the survey through the “survey monkey” website. A second and third follow-up email were sent within a month of the initial survey invitation if the participant had not completed the survey. This investigational study aimed to enroll ≥160 participants based on previous research with similar measures.^11^

### Ethical Considerations

The study protocol was refined and approved by the Institutional Review Board. All subjects provided an electronic consent. The survey was completed anonymously. The “survey monkey” web service has features that enable anonymous tracking of responses. The anonymous tracking enabled study personnel to determine which participants had not completed the survey and needed a follow-up email per the study protocol.

### Electronic Survey

Demographics, quality of life, and barriers to psychosocial support were measured through 3 sequential questionnaires.

1. Demographics questionnaire: Demographic and health history information was collected through a demographic survey. The information gathered included participant age, sex, ethnicity, marital status, diagnosis, number years since diagnosis, current disease status, family history of IBD, time in flare-up (%), surgery for IBD, employment status, and level of education.
2. Quality of life questionnaire: Quality of life was measured through the Short Inflammatory Bowel Disease Questionnaire (SIBDQ), a validated 10 item 7-point Likert scale survey designed specifically for people with IBD. It addresses the intestinal, systemic, emotional, and social aspects of quality of life.^12^ Scores range from 10 to 70 where higher scores indicate higher quality of life. A 9-point score difference was considered a clinically meaningful difference.^13^
3. Barriers to psychosocial support questionnaire: Barriers to psychosocial support was evaluated with the Perceived Barriers to Psychological Treatments scale (PBPT). The PBPT is a validated 25 item survey that encompasses 8 subscales to address different types of barriers: stigma (7 items), motivation (2 items), emotional concerns (3 items), negative evaluations of therapy (4 items), misfit of therapy to needs (4 items), time constraints (2 items), participation restriction (4 items), and availability of services (2 items). Four items cross over categories. One item investigates cost which is not factored in to a subscale score. The questionnaire ends with an open-ended question to provide comments. The PBPT scale demonstrated strong predictive validity.^14,15^

Each item on the scale presents a situation where participants answer the extent to which the situation would hinder their ability to pursue counseling. For example, participants would fill in the statement “Getting time off work to go to counseling would make it _____ for me to attend weekly counseling.” Answer options are on a 5-point Likert scale where 5 = impossible, 4 = extremely difficult, 3 = moderately difficult, 2 = slightly difficult, and 1 = not difficult at all. Both dichotomous and summed scoring methods are used. The dichotomous scoring interprets a response of a 4 or 5 as perceiving a significant barrier for that item. This method aligns with prior research suggesting that noting 1 significant barrier may be enough to prevent attending therapy. A total summed score is calculated by adding the numeric answers of each item to yield a total score ranging from 25 to 125. This method reflects the summation effect of barriers, where when more barriers are perceived the less likely the participant will be to attend counseling.^14,15^

### Statistical Analysis

Cronbach’s alpha was calculated for each of the scales to assess internal validity. Descriptive characteristics were calculated for all items on the demographic questionnaire. For the first aim of identifying the perceived barriers, the PBPT results were viewed dichotomously. The percentage of participants who answered with a 4 or 5 for each item on the PBPT was reported to quantify the frequency of a barrier. The subscale frequencies are standardized by averaging the percentages for each item in the subscale.

The second aim of assessing the relationship between perceived barriers, demographic and health characteristics, and SIBDQ scores was evaluated using summed PBPT scores. Linear regression was used to compare summed PBPT scores with demographic and health characteristics and SIBDQ scores. Comparisons were made with both the total and subscale PBPT scores. *P* < 0.05 was considered significant.

## RESULTS

### Study Population

Eight hundred seventy-nine (879) eligible patients were contacted, 210 participants consented, and 187 completed all questions. Table 1 presents the characteristics of the study population. Participants were 62% female, 89% Caucasian, and 59% in disease remission with 41% in a flare at the time of study completion.

**TABLE 1.** Characteristics of Study Population

Age (y), mean (SD)	46	(17)
Sex, n (%)		
Female	116	62%
Male	71	38%
Race/ethnicity, n (%)		
Caucasian	167	89%
African American	6	3%
Latino/Hispanic	7	4%
Asian	5	3%
Middle Eastern	1	1%
Indian/Other	1	1%
Marital status, n (%)		
Single/never married	52	28%
Married/partnered	119	64%
Separated/divorced	11	6%
Widowed	4	2%
Education, n (%)		
Less than high school	2	1%
Completed high school	31	17%
Completed college	81	43%
Schooling beyond college	73	39%
Employment status, n (%)		
Student	14	8%
Unemployed	12	6%
Part time	14	8%
Full time	111	60%
Retired	35	19%
Family history of IBD, n (%)		
No	121	65%
Yes	65	35%
Diagnosis, n (%)		
Crohn disease	96	51%
Ulcerative colitis	76	41%
Other colitis	15	8%
Time since diagnosis (y), mean (SD)	16	(14)
Current disease status, n (%)		
In remission	110	59%
Active/flaring	77	41%
Time in flare-up, n (%)		
Rarely (0%–25%)	76	41%
Sometimes (26%–50%)	61	33%
Often (51%–76%)	26	14%
Most of the time (76%–100%)	23	12%
Surgery for IBD, n (%)		
No	133	71%
Yes	54	29%
Quality of life score (SIBDQ), mean (SD)	48	(12)

Counts may not sum to 187 because of missing data.

### Internal Validity

Table 2 presents the Cronbach’s alpha for each scale. The Cronbach’s alpha for the SIBDQ was 0.81. The Cronbach’s alpha for the PBPT was 0.92 and all 8 subscales had a Cronbach’s alpha ≥0.75.

**TABLE 2.** Scale Reliability

	# Items	Cronbach’s Alpha
SIBDQ	10	0.81
PBPT	25	0.92
Stigma	7	0.85
Motivation	2	0.87
Emotional	3	0.80
Negative evaluation of therapy	4	0.83
Misfit of needs	4	0.77
Time	5	0.75
Participation restrictions	4	0.77
Availability of services	2	0.76

### Aim 1—Identifying Barriers

Table 3 presents the frequency of choosing at least 1 significant barrier overall, on each subscale, and on each question. The summed scores for each subscale and total score for the PBPT are presented for comparison. 58% of participants cited having at least 1 significant barrier. Availability of services (25%) and time (28%) were most frequently cited as a significant barrier. Least reported as barriers were stigma (14%), lack of motivation (12%), and emotional concerns (7%).

**TABLE 3.** Summary of Reported Barriers

Total PBPT score (25–125), mean (SD)	43	(14)
Total # significant barriers,* median (interquartile range)	1	(3)
Significant barrier present,* n (%)	109	58%
Stigma		
Subscale score (7–35), mean (SD)	10	(4.1)
Significant barrier present,* n (%)	25	14%
20 Discomfort with having someone see me while I am emotional	8	4%
22 Having family and/or friends know I was going to counseling	6	3%
23 Having to talk to someone I do not know about personal issues	7	4%
24 My concern about being judged by the counselor	6	3%
25 I do not think a counselor would not truly care about me	9	5%
26 Attending counseling means I cannot solve my own problems	7	4%
27 Having an insurance record of my counseling sessions	7	4%
Lack of motivation		
Subscale score (2–10), mean (SD)	3.7	(1.8)
Significant barrier present,* n (%)	22	12%
18 Lack of energy or motivation	18	10%
19 Difficulty motivating myself to do anything at all	13	7%
Emotional concerns		
Subscale score (3–15), mean (SD)	4.5	(2.1)
Significant barrier present,* n (%)	14	7%
16 Concerns about having upsetting feelings in counseling	5	3%
17 I feel that talking about upsetting issues makes them worse	8	4%
20 Discomfort with having someone see me while I am emotional	8	4%
Negative evaluation of therapy		
Subscale score (4–20), mean (SD)	6.7	(3.3)
Significant barrier present,* n (%)	36	19%
11 Having heard about or having bad or unsatisfactory experiences with counseling	9	5%
12 Distrust of counsellors	20	11%
13 I wouldn’t expect counseling to be helpful	19	10%
25 I do not think a counselor would not truly care about me	9	5%
Misfit of therapy to needs		
Subscale score (4–20), mean (SD)	6.7	(3.1)
Significant barrier present,* n (%)	33	18%
13 I wouldn’t expect counseling to be helpful	19	10%
14 Attending counseling is too self-indulgent	13	7%
21 My problems are not severe enough for counseling	17	9%
26 Attending counseling means I cannot solve my own problems	7	4%
Time constraints		
Subscale score (2–10), mean (SD)	4.7	(2.1)
Significant barrier present,* n (%)	53	28%
4 My daily responsibilities and activities	39	21%
7 Getting time off work to go to counseling	36	19%
Participation restriction		
Subscale score (4–20), mean (SD)	6.1	(2.8)
Significant barrier present,* n (%)	34	18%
1 Problems with transportation (no car/ parking, poor public transportation, etc.)	19	10%
8 Physical problems (such as difficulties walking or getting around)	4	2%
9 Physical symptoms (fatigue, pain, breathing difficulties, etc.)	6	3%
10 Serious illness which requires me to stay close to home	19	10%
Availability of services		
Subscale score (2–10), mean (SD)	4.4	(2.1)
Significant barrier present,* n (%)	46	25%
5 The lack of available counseling services in my area	23	12%
6 Not knowing how to find a good counselor	36	19%
Cost (single item)		
3 The cost of counseling	35	19%

*With score 4 (“extremely difficult”) or 5 (“impossible”).

### Aim 2—Assessing the Relationship Between Barriers and Participant Characteristics

**FIGURE 1.**
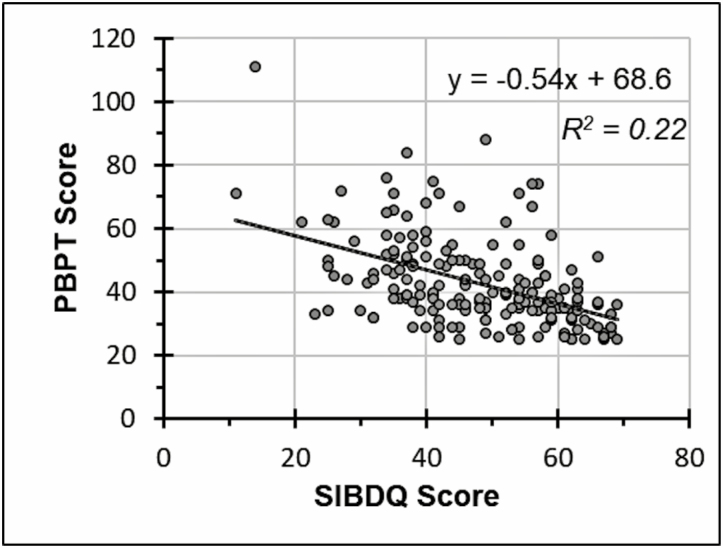
SIBDQ scores are inversely proportional to PBPT scores. Scatterplot generated from 187 participants.

The associations between participant characteristics and summed PBPT scores are presented in Table 4 for total score and Table 5 for subscale scores. Higher SIBDQ scores were associated with lower summed PBPT scores for the total score and for all 8 subscale scores. The inverse relationship between SIBDQ scores and total summed PBPT scores has an *R*^2^ of 0.22 (Fig. 1). Males compared to females had higher PBPT scores for the total score and on the misfit of needs, participation restrictions, and availability of services subscales. Not being employed full-time compared to being full-time employed was associated with higher PBPT scores on the total score and on the stigma, emotional, negative evaluation of therapy, time, and participation restriction subscales. Having Crohn disease compared to having ulcerative colitis/other colitis was associated with higher PBPT scores on the total score and on the stigma, emotional, time, and participation restriction subscales. Having disease in remission compared to having a flare was associated with higher PBPT scores on the total score and on the emotional and availability subscales.

**TABLE 4.** Predictors of Summed Total PBPT Scores (N = 187)

	Mean (SD)	MeanDif (95% CI)	*P*
Age (y)		−1 (−2.3, 0.4)	0.165
<30	47 (14)		
30–39	42 (12)		
40–59	42 (13)		
60+	41 (17)		
Sex			
Female	42 (12)		
Male	44 (17)	4.4 (0.6, 8.2)	0.025
Race/ethnicity			
Caucasian	42 (13)		
Non-Caucasian	47 (18)	3.1 (−3.0, 9.2)	0.313
Marital status			
Married/partnered	41 (13)		
Single/divorced/widowed	46 (15)	1.6 (−2.5, 5.6)	0.445
Education			0.575
High school or less	47 (16)		
College	43 (15)	−0.4 (−5.4, 4.7)	0.889
Graduate	40 (12)	−2.2 (−7.4, 3.0)	0.401
Employment status			
Full time	42 (11)		
Not full time	45 (17)	4.9 (1.0, 8.9)	0.014
Family history of IBD			
No	43 (14)		
Yes	44 (13)	3.2 (−0.5, 6.9)	0.091
Diagnosis			
Crohn disease	45 (14)		
Ulcerative colitis/other colitis	41 (13)	−6.3 (−10.6, −2.0)	0.004
Time since diagnosis (y)			
≤10	44 (15)		
>10	41 (13)	−0.5 (−2.2, 1.1)	0.537
Current disease status			
In remission	41 (14)		
Active/flaring	45 (14)	−5.8 (−10.4, −1.3)	0.013
Time in flare-up			0.148
Rarely (0%–25%)	38 (11)		
Sometimes (26%–50%)	47 (14)	4.6 (−0.2, 9.5)	0.060
Often/most of the time (>50%)	45 (16)	2.2 (−4.1, 8.4)	0.497
Surgery for IBD			
No	43 (15)		
Yes	42 (11)	−3.3 (−8.1, 1.5)	0.176
Quality of life score (SIBDQ)		−5.8 (−7.7, −3.9)	0.001
7–40	51 (15)		
41–50	42 (13)		
51+	38 (11)		

CI, confidence interval.

Mean difference for age and time since diagnosis of IBD refers to a 10-year difference; mean difference for the total SIBDQ score refers to a 10-point difference.

**TABLE 5.** Predictors of Summed Subscale PBPT Scores

	Stigma		Motivation		Emotional		Negative Evaluation		Misfit of Needs		Time		Participation		Availability	
	(N = 180)		(N = 179)		(N = 180)		(N = 180)		(N = 180)		(N = 179)		(N = 179)		(N = 178)	
Characteristic	MeanDif (95% CI)	*P*	MeanDif (95% CI)	*P*	MeanDif (95% CI)	*P*	MeanDif (95% CI)	*P*	MeanDif (95% CI)	*P*	MeanDif (95% CI)	*P*	MeanDif (95% CI)	*P*	MeanDif (95% CI)	*P*
Age (y)	−0.3 (−0.8, 0.1)	0.157	−0.2 (−0.3, 0.1)	0.101	−0.2 (−0.4, 0.0)	0.070	−0.2 (−0.5, 0.2)	0.360	0 (−0.3, 0.4)	0.791	−0.2 (−0.4, 0.1)	0.101	0.1 (−0.2, 0.4)	0.401	0 (−0.2, 0.2)	0.774
<30																
30–39																
40–59																
60+																
Sex																
Female																
Male	1 (−0.3, 2.2)	0.138	0.1 (−0.4, 0.6)	0.611	0.3 (−0.3, 1.0)	0.266	0.8 (−0.2, 1.7)	0.129	1.2 (0.3, 2.2)	0.012	0.2 (−0.4, 0.9)	0.518	1 (0.2, 1.8)	0.012	0.6 (0.0, 1.2)	0.049
Race/ethnicity																
Caucasian																
Non-Caucasian	−0.3 (−2.3, 1.7)	0.765	0.4 (−0.4, 1.2)	0.318	−0.1 (−1.1, 0.9)	0.811	0.1 (−1.5, 1.6)	0.949	0.3 (−1.2, 1.8)	0.729	0.3 (−0.7, 1.3)	0.533	0.9 (−0.4, 2.1)	0.166	1 (0.1, 2.0)	0.029
Marital status																
Married/partnered																
Single/divorced/widowed	0.1 (−1.2, 1.4)	0.887	0.2 (−0.4, 0.7)	0.533	0.1 (−0.5, 0.7)	0.769	0.6 (−0.4, 1.6)	0.255	0 (−1.0, 1.0)	0.957	0.1 (−0.5, 0.8)	0.674	0.7 (−0.2, 1.5)	0.118	0 (−0.6, 0.7)	0.905
Education		0.381		0.148		0.387		0.604		0.793		0.826		0.863		0.559
High school or less																
College	0.4 (−1.2, 2.1)	0.604	−0.4 (−1.0, 0.3)	0.278	−0.1 (−0.9, 0.7)	0.754	−0.5 (−1.8, 0.8)	0.459	−0.3 (−1.6, 0.9)	0.617	0 (−0.9, 0.9)	0.999	0.1 (−0.9, 1.1)	0.835	0.2 (−0.6, 0.9)	0.679
Graduate	−0.5 (−2.2, 1.2)	0.583	−0.7 (−1.4, 0.0)	0.055	−0.5 (−1.3, 0.3)	0.241	−0.7 (−2.0, 0.6)	0.316	−0.4 (−1.7, 0.8)	0.496	0.2 (−0.7, 1.1)	0.664	−0.1 (−1.2, 0.9)	0.835	−0.2 (−1.0, 0.6)	0.677
Employment status																
Full time																
Not full time	1.8 (0.5, 3.2)	0.006	0.5 (−0.1, 1.0)	0.074	0.6 (0.0, 1.3)	0.048	1.5 (0.5, 2.5)	0.004	0.6 (−0.4, 1.6)	0.236	−1 (−1.6, −0.3)	0.004	1.5 (0.6, 2.3)	0.001	0.3 (−0.3, 0.9)	0.329
Family history of IBD																
No																
Yes	0.9 (−0.4, 2.1)	0.167	0.1 (−0.4, 0.6)	0.813	0.1 (−0.5, 0.7)	0.774	1 (0.1, 2.0)	0.035	0.7 (−0.2, 1.6)	0.135	0.6 (0.0, 1.3)	0.042	0 (−0.8, 0.7)	0.942	0.1 (−0.4, 0.7)	0.636
Diagnosis																
Crohn disease																
Ulcerative colitis/other colitis	−1.5 (−3.0, −0.1)	0.036	−0.3 (−0.9, 0.3)	0.290	−0.7 (−1.4, 0.0)	0.049	−1.1 (−2.2, 0.0)	0.057	−1.1 (−2.2, 0.1)	0.053	−0.8 (−1.5, −0.1)	0.027	−1 (−1.9, −0.1)	0.023	−0.5 (−1.2, 0.2)	0.136
Time since diagnosis (y)																
≤10																
>10	−0.2 (−0.7, 0.4)	0.530	−0.1 (−0.3, 0.1)	0.498	−0.1 (−0.3, 0.2)	0.549	−0.1 (−0.5, 0.3)	0.751	−0.3 (−0.7, 0.1)	0.201	0 (−0.3, 0.3)	0.941	0.1 (−0.3, 0.4)	0.691	−0.1 (−0.4, 0.1)	0.284
Current disease status																
In remission																
Active/flaring	−1.4 (−2.9, 0.2)	0.079	−0.2 (−0.8, 0.4)	0.474	−0.8 (−1.5, −0.1)	0.028	−0.6 (−1.8, 0.6)	0.309	−1.1 (−2.2, 0.1)	0.069	−0.7 (−1.4, 0.1)	0.087	−0.7 (−1.6, 0.3)	0.166	−0.9 (−1.6, −0.2)	0.011
Time in flare-up		0.442		0.976		0.113		0.124		0.304		0.367		0.196		0.392
Rarely (0%–25%)																
Sometimes (26%–50%)	1 (−0.6, 2.6)	0.219	−0.1 (−0.7, 0.6)	0.855	0.8 (0.0, 1.5)	0.046	0.9 (−0.3, 2.1)	0.161	0.9 (−0.3, 2.1)	0.126	0.6 (−0.2, 1.4)	0.158	0.8 (−0.2, 1.8)	0.128	0.5 (−0.2, 1.2)	0.185
Often/most of the time (>50%)	0.5 (−1.6, 2.5)	0.670	0 (−0.8, 0.8)	0.996	0.3 (−0.7, 1.3)	0.509	−0.3 (−1.9, 1.3)	0.695	0.6 (−1.0, 2.1)	0.470	0.4 (−0.7, 1.4)	0.468	1.1 (−0.2, 2.4)	0.093	0.2 (−0.7, 1.2)	0.621
Surgery for IBD																
No																
Yes	−1.1 (−2.7, 0.5)	0.177	0.7 (0.1, 1.3)	0.031	−0.7 (−1.5, 0.0)	0.053	−0.7 (−1.9, −0.5)	0.272	−0.7 (−1.9, 0.5)	0.229	−0.9 (−1.7, −0.1)	0.031	−0.6 (−1.6, 0.4)	0.234	0 (−0.8, 0.7)	0.897
Quality of life score (SIBDQ)	−1.1 (−1.7, −0.4)	0.001	−0.7 (−1.0, −0.5)	0.001	−0.6 (−1.0, −0.3)	0.001	−1 (−1.5, −0.5)	0.001	−0.8 (−1.2, −0.3)	0.002	−0.4 (−0.7, −0.1)	0.017	−0.9 (−1.3, −0.5)	0.001	−0.9 (−1.2, −0.6)	0.001
7–40																
41–50																
51+																

CI, confidence interval.

Mean difference for age and time since diagnosis of IBD refers to a 10-year difference; mean difference for the total SIBDQ score refers to a 10-point difference.

Participant characteristics that were not significantly associated with summed PBPT scores include age, race, marital status, having a family history of IBD, years since diagnosis, the percent of time in a flare-up, and having had surgery for IBD.

### Aim 3—Institutional Suggestions

There were 59 optional free response comments submitted. They were categorized as a suggestion (2), an elaboration on barriers (40), study feedback (4), and noncontributory (16), with 3 comments overlapping categories. Suggestions offered include proposing an IBD-specific counselor where “knowledge about the disease would be beneficial to the counseling relationship” and at-home counseling sessions.

## DISCUSSION

Psychological disorders are a common comorbidity among people with IBD that negatively impact quality of life. While psychotherapy has been shown to be a valued and effective treatment, people do not always pursue the therapy. This study sought to identify the barriers to psychotherapy. The study also aimed to identify relationships between barriers and demographical and health characteristics. This knowledge will help providers to use valuable patient care time more effectively.

### Barriers Among Patients With IBD

This is the first study that has used the PBPT scale among patients with IBD. The internal validity of the PBPT in the present study was acceptable-excellent. The first aim was successfully completed. The percentage of participants identifying at least 1 significant barrier was 58%. This result is similar to a previous study in a non-IBD population where the author’s found that 55% of participants identified at least 1 significant barrier.^14^ The previous study also found that when comparing participants with and without depression, the rate of identifying at least 1 significant barrier increased to 78% from 49% in patients with depression.^14^ It is possible that a similar rate increase among people with depression exists in the IBD population.

The leading barriers identified in the present study were time constraints and availability of services. Time constraints have been found to be particularly predictive of following up with psychotherapy.^14^ While it did not qualify for its own subscale, the item that assessed cost barriers was the item most frequently endorsed as a barrier. Cost has also been cited as the greatest barrier in other research.^14,16^ Interestingly, stigma was not endorsed as a significant barrier. This differs from a prior study among people with depression where stigma in addition to cost and knowledge about how to find help (availability of services) were leading barriers.^16^ The present study suggests that people with IBD share the barriers of cost, time, and a lack knowledge about availability of services but do not share the stigma barrier with people who do not have IBD.

The second aim of the study was to investigate the relationship between barriers, participant characteristics, and quality of life. Quality of life was found to have an inverse relationship with the perception of barriers. This consistent correlation may be because some of the factors that enhance quality of life, like overall disease severity, are also the ones that reduce barriers.^13^ Other characteristics associated with increased barriers were being male, not being full-time employed, having Crohn disease, and having disease in remission. Despite the small subgroup sample sizes, the consistent significant association in the overall and subscale scores affirms that participants with these particular characteristics may be at increased risk of perceiving greater barriers. There was not a consistent association between perceived barriers and age, race, relationship status, level of education, time since diagnosis, family history of IBD, percent of time in flare-up, or prior surgeries for IBD. These results differ from prior research in the non-IBD population that found higher total PBPT scores among non-Caucasian participants, participants without partners, and younger participants and no difference in total PBPT score based on gender.^14^

### Evaluation of Study

The study has several limitations. First, the sample size does not allow for sufficiently powered subgroup analysis. Modest though important differences in significant subscale categories and participant characteristics may have been be overlooked. When significant results were found in the subgroup and subscale analyses, caution was taken to emphasize only the consistent, significant trends in the discussion. Additionally, the present study does not account for preexisting psychiatric conditions. The survey initially included a question on psychiatric history but eliminated the data as the survey format does not ensure the accurate or complete collection of psychiatric history. In future research it may be worthwhile including a question of psychiatric conditions as well as a Patient Health Questionnaire (PHQ). A PHQ scores have been used in prior research using the PBPT.^4,14,15^ A PHQ score would help explore how depression severity and mood symptoms affect perceived barriers.^3,6^

Another limitation is that the survey did not inquire about current or past psychotherapy experiences. This was omitted as the PBPT accounts for the influence of past psychotherapy experiences (item 11 displayed in Table 2). Further, the PBPT scale has been found to have strong predictive validity.^14,15^ Other limitations include a sampling bias where only patients with an email listed in their electronic medical record were contacted and a participation bias favoring those intrigued by the topic of mental health and IBD. Additionally, the demographic and health characteristics of the participants are unevenly distributed being predominantly made up of Caucasians, females, full-time workers, people in a relationship, and those who received higher education. Individuals from groups less represented in this study may be at increased risk of having greater perceived barriers.

An important strength of the study was that it was entirely anonymous. Providing personal information regarding psychological and gastrointestinal illnesses relative to other illnesses may cause participants to feel particularly vulnerable and reluctant to share honest information. The electronic survey allowed the study to be conducted while maintaining anonymity. Further, participants were able to complete the survey in the location of their choosing, allowing for privacy. Additionally, the internal validity of the survey questions was optimized by the use of unmodified, validated questionnaires.

### Suggestions for Reducing Barriers to Psychotherapy

The third and final aim of this study is to provide institutional suggestions on ways to reduce barriers to mental healthcare. First, knowing the characteristics associated with having barriers and knowing what the most common barriers are allows providers to identify at-risk patients and plan their visits accordingly. The present study suggests that males and those not full-time employed, among others, may be at increased risk of perceiving greater barriers. Regarding the most common barriers, the availability of services barrier is based on people perceiving a lack of available counseling services in the area and not knowing how to find a good counselor (2 items from the PBPT). Patients would benefit from their care provider helping them to navigate the services available. To enhance this process, a gastroenterology office can establish a network of reliable therapists. Referring patients to a therapist already trusted and with experience working with people with IBD would alleviate this barrier—and address the suggestion offered by one of the participants. To establish this network, providers can learn about resources from patients who have had successful therapist relationships, consult with colleagues in other departments who have found a trusted therapist or reliable network, create a behavioral health team for their department by hiring therapists with experience and interest caring for patients with gastrointestinal illnesses, provide resources such as weblinks or pamphlets to patients to help them search for a therapist whose practice aligns with their needs regarding hours of availability, location, and telehealth capabilities.

For people who travel further distances for their care, telehealth psychotherapy visits may help to decrease this barrier. Telehealth psychotherapy has been found to be no less effective than non-telehealth psychotherapy for depression.^17^ The barrier of having time constraints could also be in part minimized by telehealth services.

When it comes to the cost barrier, solutions are more difficult. Informal, low cost, or free online discussion groups and other resources for support exist, but there is currently no evidence on how they affect disease outcomes or quality of life.^18^ Like other formal, long-term therapies, people rely on their health insurance to help cover costs. The different healthcare plans cover mental health support to various extents, but all leave a burden on the patient and limit the availability of services. Increasing the number of psychotherapists familiar with caring for people with IBD, providing telehealth psychotherapy, and diminishing costs of psychotherapy and mental healthcare services all rely on changes in the healthcare system at large. This study starts to elicit those changes by identifying their need.

## CONCLUSIONS

This is the first study to identify the perceived barriers to psychotherapy among the IBD population and to delineate associated demographic or health characteristics of people who are at an increased risk of perceiving greater barriers. Cost, time restraints, and the availability of services were found to be the leading perceived barriers. Additionally, having greater perceived barriers was found to be associated with a lower quality of life. Further research should be conducted focusing on groups underrepresented in this study. Additionally, gastroenterology offices should develop a network of reliable psychotherapists who are available to people with IBD. Finally, successful innovations in psychotherapy, like telehealth visits, should continue to be researched and promoted.

## Data Availability

All data referenced are available in tables and figures within the manuscript.

## Notes

Funding: None.
